# [Retracted] Inhibition of microRNA‑19b promotes ovarian granulosa cell proliferation by targeting IGF‑1 in polycystic ovary syndrome

**DOI:** 10.3892/mmr.2023.13057

**Published:** 2023-07-24

**Authors:** Zhuohui Zhong, Fang Li, Yingying Li, Shuang Qin, Canliang Wen, Yiyuan Fu, Qing Xiao

Mol Med Rep 17: 4889–4898, 2018; DOI: 10.3892/mmr.2018.8463

Following the publication of this paper, it was drawn to the Editor's attention by a concerned reader that the colony formation assay data (or portions of the data thereof) shown in Figs. 2C and 5G were strikingly similar to data that had appeared in different form in other articles by different authors at different research institutes. Owing to a general lack of confidence in the presented data, and due to the fact that the contentious data in the above article may have already been published, prior to its submission to *Molecular Medicine Reports*, the Editor has decided that this paper should be retracted from the Journal. The authors were asked for an explanation to account for these concerns, but the Editorial Office did not receive a reply. The Editor apologizes to the readership for any inconvenience caused.

